# Genetic Engineering and Biosynthesis Technology: Keys to Unlocking the Chains of Phage Therapy

**DOI:** 10.3390/v15081736

**Published:** 2023-08-14

**Authors:** Sixuan Lv, Yuhan Wang, Kaixin Jiang, Xinge Guo, Jing Zhang, Fang Zhou, Qiming Li, Yuan Jiang, Changyong Yang, Tieshan Teng

**Affiliations:** 1 School of Nursing and Health, Henan University, Kaifeng 475004, China; 2 Institute of Biomedical Informatics, School of Basic Medical Sciences, Henan University, Kaifeng 475004, China

**Keywords:** genetic engineering, biosynthesis technology, phage therapy, antibiotics

## Abstract

Phages possess the ability to selectively eliminate pathogenic bacteria by recognizing bacterial surface receptors. Since their discovery, phages have been recognized for their potent bactericidal properties, making them a promising alternative to antibiotics in the context of rising antibiotic resistance. However, the rapid emergence of phage-resistant strains (generally involving temperature phage) and the limited host range of most phage strains have hindered their antibacterial efficacy, impeding their full potential. In recent years, advancements in genetic engineering and biosynthesis technology have facilitated the precise engineering of phages, thereby unleashing their potential as a novel source of antibacterial agents. In this review, we present a comprehensive overview of the diverse strategies employed for phage genetic engineering, as well as discuss their benefits and drawbacks in terms of bactericidal effect.

## 1. Introduction

The World Health Organization (WHO) has proposed that antimicrobial resistance is one of the greatest threats to global public health in the 21st century. According to WHO, approximately 700,000 individuals succumb to antibiotic resistance annually [1,2]. Furthermore, it is estimated that by 2050, ten million deaths will be attributed to drug-resistant bacterial infections [3]. Meanwhile, the increasing prevalence of antibiotic resistance suggests the end of the golden era of antibiotics and the possibility of entering a post-antibiotic era, where we face a world lacking efficient antimicrobial agents [4,5,6]. 

Bacteriophages (phages), also known as bacterial viruses, exhibit a high specificity towards particular bacterial species, with fewer off-target effects on intestinal microbiota compared to antibiotics [7,8,9]. Although the first successful application of phage therapy was reported nearly a century ago, it has recently regained attention due to the escalating threat of antibiotic resistance. Despite the potential of phages as antimicrobial agents [10], several major concerns remain with regard to their clinical applications, such as the emergence of phage-resistant bacteria [11], the detrimental inflammatory response [12], and the physical barrier formed by biofilm [13]. However, genetic engineering and biosynthesis technology allow for the modification of phages to expand their host range and enhance their therapeutic potential, offering promising solutions to these challenges [14]. For example, phages can be engineered to express enzymes that degrade bacterial biofilms, which are often resistant to antibiotics and phages. Biosynthesis technology enables the production of phages on a large scale, which is essential for clinical applications. In addition, genetic engineering and biosynthesis technology can be combined to create synthetic phages with tailored properties. For instance, synthetic phages can be designed to target specific bacterial strains or to carry therapeutic payloads such as antibiotics or immunomodulators. Moreover, the growing availability of complete phage genome sequences in public databases, coupled with advances in understanding the structure of phage components and phage-host bacterial interactions, has facilitated the targeted engineering of phages [15]. 

Genetic engineering and biosynthesis technology hold great promise for advancing phage therapy and overcoming the challenges associated with bacterial infections. This paper aims to present a comprehensive review of the latest advancements in phage engineering and biosynthesis technology, as well as their diverse applications in the treatment of bacterial infections.

## 2. Phage Engineering and Biosynthesis Strategies

Recent advances in phage engineering and biosynthesis have led to the development of phage-based therapeutics with has improved efficacy and safety profiles. These include host-mediated homologous recombination, in vivo recombineering, BRED, yeast-based assembly of phage genomes, L-form bacteria, and CRISPR-Cas. Phage engineering and biosynthesis strategies hold great promise for the development of novel antibacterial agents to combat antibiotic-resistant bacteria.

### 2.1. Host-Mediated Homologous Recombination

**Figure 1:**
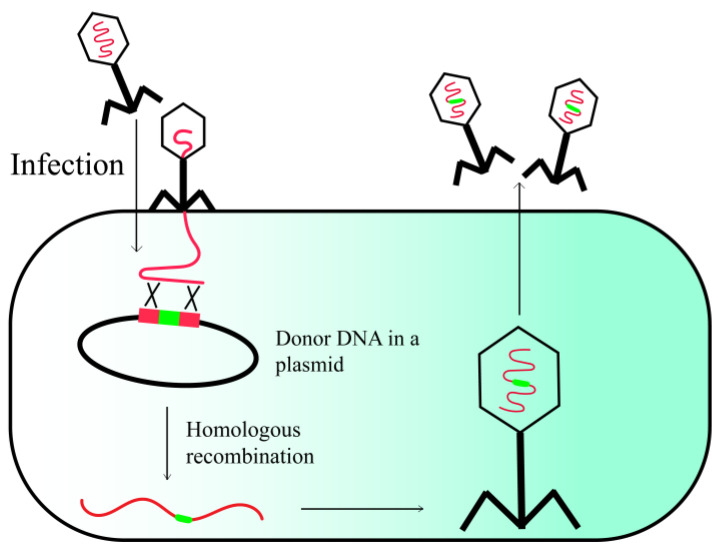
Phage engineering via homologous recombination.

Homologous recombination (HR) is a highly versatile strategy for phage genome engineering, which can occur between two DNA fragments that share only limited regions of homology (Figure 1). To execute HR, exogenous DNA segments containing flanking regions homologous to the phage genome sequence are initially cloned into a vector [16]. The homologous regions dictate the integration site of exogenous DNA segments within the phage genome. The host strain carrying the donor vector is subsequently infected with the engineered phage. In vivo, homologous recombination (HR) occurs between the vector and the phage genome, facilitated by host recombinases, such as RecA. This process enables the integration of foreign DNA fragments into the phage genome, which are ultimately packaged within the phage particles [17]. Recent studies have demonstrated that retrons possess the ability to function as a recombination template, without the need for lengthy homologous flanking sequences. This feature facilitates a rapid and uncomplicated cloning procedure, although the insertion of large fragments may be limited by the restricted size of the homologous domain in the retron, which typically spans 75 base pairs (75 bp) [18,19]. However, one significant drawback of engineering methods based on homologous recombination is the relatively low efficiency of recombination, which necessitates time-consuming and labor-intensive screening methods to identify recombinant phages. This limitation arises from the inability to employ selectable markers, such as antibiotic resistance genes, during the lytic growth phase of phages. To streamline the screening process, it is feasible to integrate marker genes that enable a targeted selection for mutated phages or to employ a subsequent counter-selection technique to remove the wild-type phages (refer to the subsequent sections for more information).

### 2.2. In Vivo Recombineering 

**Figure 2:**
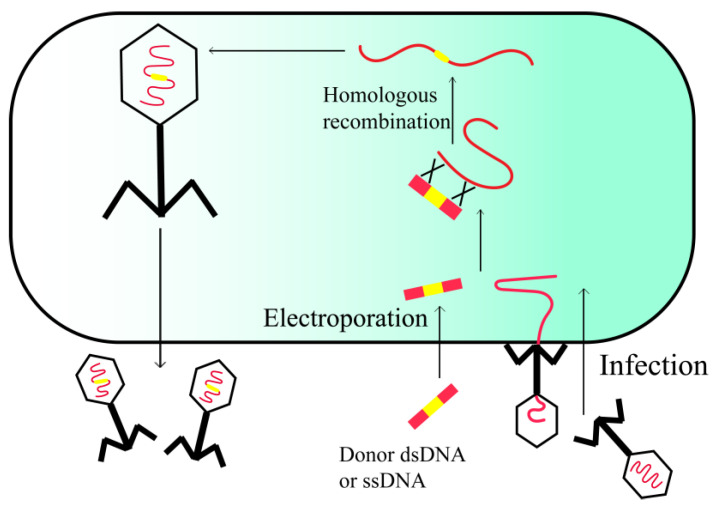
In vivo recombineering of phage.

HR is a rare occurrence in most organisms, and attaining the desired recombinant through endogenous recombination machinery is frequently challenging. To circumvent this issue, an improved editing technology termed recombineering has been devised. It employs the recombination system harbored by phage genomes to increase the frequency of HR, thereby enabling the generation of gene knockouts, deletions, and point mutations (Figure 2) [20]. 

Recombineering relies on linear donor DNA and heterologous proteins, including Gam, Bet, and Exo, which are expressed from the phage genome. These proteins protect linear dsDNA from intracellular degradation and promote recombination between the linear donor DNA and the injected phage genome. This process increases the frequency of recombination and reduces the length of homologous regions to as little as 50 bp [21]. The enhanced accessibility of phage genome sequences has greatly facilitated the convenience of recombineering, as it is only viable to recombine a phage with a known genome sequence [22].

### 2.3. BRED 

**Figure 3:**
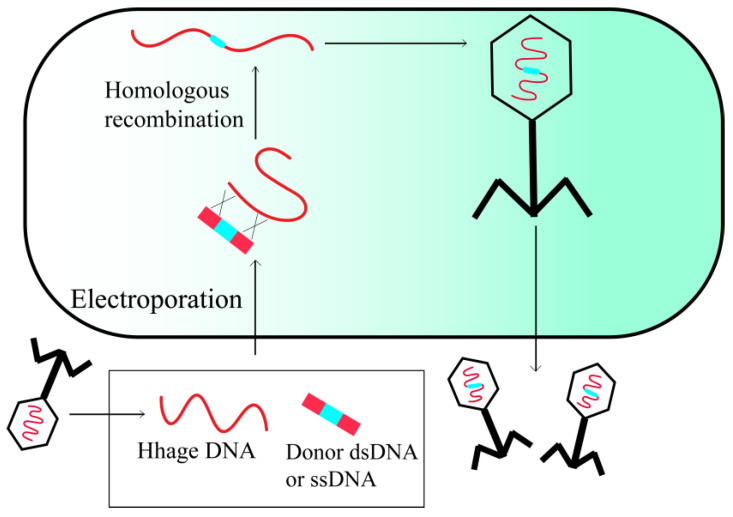
Phage recombineering of electroporated DNA.

Bacteriophage recombineering with electroporated DNA (BRED) is a valuable technique for genetically modifying lytic phages. This method entails the electroporation of linearized phage genome fragments and homologous DNA into phage-sensitive bacterial cells harboring plasmids that express HR-enhancing proteins, such as RecE/RecT-like proteins (Figure 3) [23]. Because the success rate of BRED is relatively high (10% in *M. smegmatis*), recombinant phages can be retrieved through PCR-based plaque recovery following cell lysis and infection of susceptible hosts. The benefits of BRED include the absence of a need for constructing intricate cloning systems and a selectable marker, as well as the ability to introduce mutations into any region of the phage genome [24]. BRED offers the potential to produce phages that have undergone editing without recombination, thereby excluding them from the classification of genetically modified organisms. This enables regulatory agencies to more readily approve the use of modified phages for therapeutic purposes, as evidenced by the recent authorization of the first engineered phage therapy in the United Kingdom [25].

At present, the progress of BRED is primarily hindered by challenges in attaining sufficiently high transformation efficiencies when working with the extensive genomes of phages in various host organisms [26]. In order to address these constraints, alternative approaches to BRED have been utilized, such as the utilization of bacteriophage recombineering with infectious particles (BRIP) [27]. In this method, the synthetic DNA substrate containing the desired modification is introduced into the bacteria through electroporation followed by infection of the bacteria with the phage, as opposed to the conventional approach of electroporating the bacteria with phage DNA.

### 2.4. Yeast-Based Assembly of Phage Genomes

**Figure 4:**
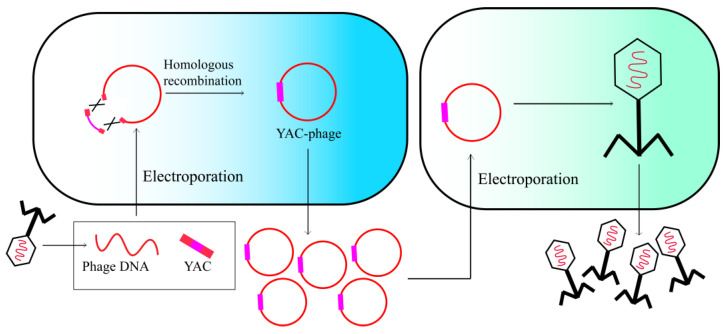
Yeast-based assembly of phage genomes.

Phage genome modification can be achieved by utilizing a yeast cell as an alternative host for phage assembly. The intrinsic capacity of *Saccharomyces cerevisiae*, a yeast species, to recombine linear double-stranded DNA fragments into a single genome facilitates genetic modifications and empowers the generation of engineered phages [28]. To assemble the complete phage genome in yeast, PCR amplification is conducted with homologous termini that are retained with at least 30 bp in length. The initial and final genome fragments are amplified using primers that contain homologous sequences with a yeast artificial chromosome (YAC). All amplified segments of the genome and YAC are subsequently transformed into *S. cerevisiae*, where gap repair enables a recombination-mediated joining of all phage genome segments (Figure 4). After purifying the recombinant vector, the phage genome can be assembled to generate phage particles upon transformation into the host bacteria. The resulting plaques are subsequently selected and sequenced to confirm the successful incorporation of the desired mutations [29]. This phage engineering strategy has been utilized to genetically modify multiple phage genomes, such as T3 and φX174 coliphages, as well as *Klebsiella* phage K11 [28,30,31]. 

The utilization of YAC-assisted assembly for modified genomes has proven to be highly efficient. However, this approach is not devoid of limitations, as certain phages may possess repetitive sequences at their termini that could result in a phage excision from the vector during recombination [32]. Nevertheless, this challenge can be overcome by incorporating selective markers for yeast within the phage genome, which is a promising approach. In addition, the process of rebooting phage genomes has traditionally relied on the transformation of host bacteria, which limits the potential application of this engineering strategy to bacteria that are highly transformable [33]. As an alternative, it may be possible to reboot assembled phages through alternative methods such as the use of bacterial L-forms and cell-free techniques.

### 2.5. L-Form Bacteria

**Figure 5:**
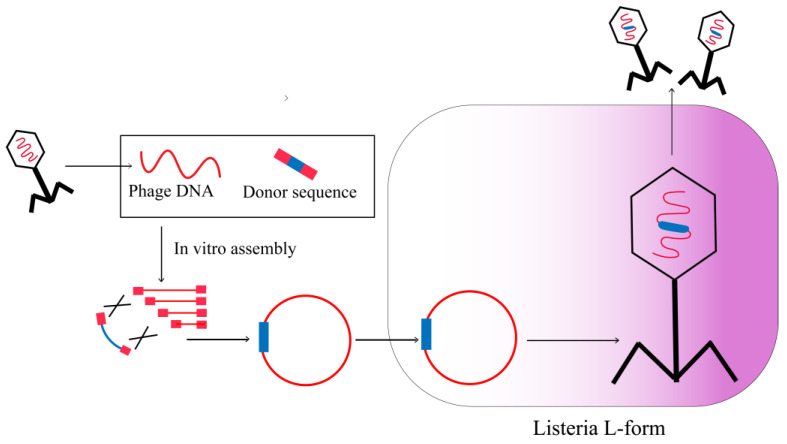
Synthetic phage genomes in L-form bacteria.

The limited ability of Gram-positive bacteria to undergo transformation poses a significant obstacle in the reactivation of engineered phages that target these hosts. However, this limitation can be overcome by substituting the robust cell walls of Gram-positive hosts with L-form bacteria, thereby enabling the reactivation of the synthetically constructed phage genome. L-form bacteria are cell-wall-deficient organisms that are capable of undergoing transformation with macromolecular DNA, as well as replicating and transcribing newly introduced genetic molecules. Drawing on these advantages, L-form bacteria were assessed as potential hosts for rebooting the synthetic genomes of phages (Figure 5). Furthermore, Kilcher et al. demonstrated the ability to reboot various Gibson assembled or wild-type phage genomes in L-form *Listeria monocytogenes* or related Gram-positive hosts. L-type bacteria release phage particles via hypoosmotic lysis, which enables the phages to infect specific bacterial hosts for reproduction. The L-forms of *Listeria monocytogenes* have been found to possess the potential to serve as a platform for the reactivation of *Bacillus* and *Staphylococcus* phages, indicating the intergeneric applicability of L-form cells in phage genome engineering [34]. This discovery is of significant interest and may have important implications for the development of novel strategies for phage-based therapies.

The ability to reboot phage genomes in bacterial L-forms has been observed to be largely unaffected by various factors such as the viral lifestyle, morphology, DNA packaging strategy, genome ends (including cohesive ends, terminally redundant, and all double-stranded DNA), and size [34]. This finding provides a valuable engineering platform for a diverse array of phages. Although the rebooting of engineered phage genomes in L-forms has thus far only been observed in Gram-positive cells, the potential for generating L-forms of Gram-negative bacteria implies that they could also serve as phage rebooting mechanisms [35]. However, it remains uncertain whether L-forms can be effectively produced for all types of bacteria.

### 2.6. CRISPR-Cas

**Figure 6:**
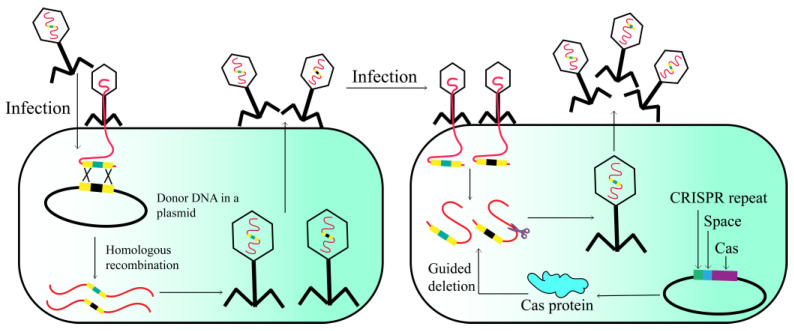
Phage genome engineering with CRISPR-Cas13a.

The CRISPR-Cas system is a naturally occurring adaptive defense mechanism present in numerous prokaryotes, which confers sequence-specific protection against invasive nucleic acids [36,37,38]. The CRISPR-Cas system comprises two genetic components: Cas proteins and the CRISPR array [39,40,41]. The type I-E CRISPR-Cas system has recently been utilized as a counter-selection mechanism for engineered T7 phages (Figure 6). The phage underwent homologous recombination-mediated editing to excise the dispensable gene 1.7 [42]. The strategy selectively eliminated nonrecombinant phage genomes harboring gene 1.7, while sparing the recombinant phage genomes devoid of this gene. Similarly, the CRISPR/Cas II-A system can also be utilized for in vivo editing of the phage 2972 genome, encompassing point mutations, gene deletions, and DNA exchange [37]. Given the successful genetic manipulation demonstrated in the above results, the CRISPR/Cas strategy holds promise for application to other phage genomes.

The utilization of CRISPR-Cas-based phage engineering techniques is constrained to bacteria that possess a well-defined native CRISPR-Cas system or have the ability to undergo transformation in order to facilitate the expression of an operational heterologous CRISPR-Cas system. This can pose a significant constraint for the manipulation of bacteriophages targeting bacteria that lack genetic manipulability. Furthermore, phages have developed various mechanisms to resist targeting by CRISPR-Cas, including the concealment of their DNA through covalent modifications of nucleotides or the utilization of anti-CRISPR proteins (Acrs) [43]. The extensively studied phage T4 of *E. coli* has been found to exhibit glucosyl DNA hypermodifications as well as DNA recombination and repair mechanisms. These mechanisms serve to safeguard the phage against the DNA-targeting type I and II CRISPR-Cas systems, thereby leading to a diminished efficacy in counter-selecting the wild-type T4 [44,45].

## 3. Application of Engineering Phage

Phage therapy has emerged as a promising approach to address the global rise of antibiotic resistance among bacterial pathogens. Advancements in biosynthesis and genetic engineering technologies have greatly facilitated phage genome engineering. Significant progress has been made in engineering phages to restore the sensitivity of drug-resistant bacteria, reduce the minimum inhibitory concentration (MIC) of antibiotics, target the deletion of essential genes of host bacteria, and provide crucial therapies for patient treatment (Table 1). Notably, these advancements hold immense potential in combating antibiotic resistance and improving patient prognosis. 

### 3.1. Enhancing Bactericidal Activity 

The bactericidal efficacy of natural phages varies depending on the specific phage and can be enhanced through the development of genetically modified phages. A phage K1F was genetically modified using the homologous recombination technique to incorporate fluorescence and facilitate the expression of epidermal growth factor (EGF) derived from the ErbB family of tyrosine kinases [46]. The modified phage K1F bearing EGF, demonstrated an increased ability to enter human cells and exhibited improved effectiveness in eradicating intracellular *E. coli* EV36. Distinctive trafficking pathways between the two phages were also observed: K1F-GFP-EGF entered cells through the endolysosomal pathway by inducing the EGF receptor (EGFR), while K1F-GFP entered cells and underwent degradation through LC3-assisted phagocytosis. This enabled K1F-GFP-EGF to accumulate rapidly within different human cell lines, thereby enhancing its efficiency in locating its intracellular host. 

### 3.2. Restoring the Sensitivity of Drug-Resistant Bacteria to Antibiotics

RNA-guided nucleases (RGNs), derived from Type II CRISPR-Cas systems, are programmable endonucleases that enable precise genome editing. RGNs comprise two essential components: a guide RNA (gRNA) of approximately 100 nucleotides, which utilizes 20 variable nucleotides at its 5′ end to form base pairs with a specific genomic DNA sequence, and a nuclease (e.g., Cas9 endonuclease) that cleaves the target DNA [47]. Several studies have demonstrated that modified phages overexpressing RGNs can restore the sensitivity of drug-resistant bacteria to antibiotics [48,49,50]. For instance, Ido Yosef and colleagues designed a temperate phage to transfer the effective RGNs into the genome of antibiotic-resistant bacteria [51]. The gRNA sequences were initially designed to target conserved regions of the resistance genes ndm-1 and ctx-M-15. Subsequently, the designed gRNA and Cas9 endonuclease encoding gene were introduced into λ prophage via homologous recombination. As a result, the resistance gene in *E. coli* lysogenized with the engineered λ prophage was deleted, resulting in a restored antibiotic sensitivity. In another study, Edgar R.’s group employed homologous recombination to introduce a streptomycin-sensitive gene into a phage genome [52]. This engineered phage treatment effectively restored antibiotic sensitivity in streptomycin-resistant *E. coli*, resulting in a significant reduction of the MIC of antibiotics from 100 to 12.5 mg/mL. The same strategy restored the sensitivity of *E. coli* to naphthyridic acid, resulting in a twofold reduction in the MIC value.

These strategies could be utilized to treat hospital surfaces and hand sanitizers, specifically targeting the skin microbiota of medical personnel. Unlike antibiotics and disinfectants, which promote the growth of resistant pathogens, this proposed treatment enriches and selects for susceptible pathogens. Additionally, these strategies promote the growth of pathogens that are unable to acquire or transfer resistance determinants horizontally, potentially reducing the spread of antibiotic resistance. The enriched susceptible population may also prevent the establishment of newly introduced resistant pathogens by outcompeting them for their ecological niche.

### 3.3. Altering Phages’ Host Range

One major limitation of phage-based applications is their narrow and specific host range. The natural host range of phages is insufficient to cover all pathogenic microbial strains. To circumvent this challenge, genetic engineering techniques have been adopted to expand or alter the host range of phages [53]. Several reports have demonstrated that the host specificity of phages is determined by their tail fiber [54]. To broaden the host range of phages, researchers employed a homologous recombination technique to rationally replace the tail fiber encoding genes of phage T2 with their homologous counterparts from phage IP008 [55]. The chimeric phage generated exhibited an expanded host range compared to the original T2 phage, while maintaining an equivalent lytic activity [56]. In a similar study, the gene encoding the tail fiber (gp69) in phage PaP1 was substituted with the homologous gene (gp84) from phage JG004 via HR, resulting in the creation of an engineered phage that could induce plaque formation in the bacterial host of phage JG004 [57]. In another study, the host range of the filamentous phage fd was expanded through the incorporation of a receptor-binding domain from the phage IKe into the N-terminus of protein G3p [58]. Similarly, phage fd could be engineered to recognize *Vibrio cholerae* by fusing the minor coat-encoding gene pIII from fd with a sequence of the *orfU* gene from CTXφ, another filamentous phage [59]. However, these strategies are constrained by the need to modify tail components that recognize host receptors, which are specific to known phages. These methods offer a versatile tool for rapidly altering and broadening the host range of bacteriophages. With the acquisition of a greater number of suitable homologous sequences, future clinical applications for screening phages of specific clinical isolates can be developed. Furthermore, these methods may enable a direct modification of bacteriophage genomes to expand or alter their host ranges. 

### 3.4. Increasing the Cleavage of Biofilm

Bacterial growth within biofilms is frequently associated with the pathogenesis of numerous clinically important infections [60]. Biofilms pose a formidable challenge for eradication due to their inherent resistance to antimicrobial agents, such as phages and antibiotics. This phenomenon is often attributed to the extracellular polymeric substances (EPS) of biofilm, which limit the diffusion of molecules. To address this issue, *E. coli*-specific phage T7 was genetically modified to incorporate the biofilm-degrading enzyme dispersin B (DspB) during phage infection [61]. DspB exerts its action via the aminohydrolysis of β-1, 6-Nacetyl-D-glucosamine, thereby disrupting the biofilm’s formation and integrity. This engineered T7 phage exhibited a significant increase of 4.5 orders of magnitude in biofilm reduction after a 24 h treatment compared to its nonengineered counterpart, while the control group only showed a reduction of 2.5 orders of magnitude [61]. These findings indicated that the expression of DspB was crucial for elevating the efficacy of biofilm removal in the engineered virus, while the control group without DspB expression failed to achieve the same effect.

Acyl-homoserine lactone (AHL) is a well-known quorum-sensing signal that plays a crucial role in the formation of biofilm. The activity of AHLs can be effectively inhibited by AHL-lactonases, which catalyze the cleavage of the lactone bond in AHLs, thereby preventing biofilm formation [62]. Accordingly, the gene encoding AHL-lactonase (aiiA) from *Bacillus anthracis* was cloned into the T7 phage, resulting in the creation of an engineered phage named T7aiiA. The biofilm formation was significantly inhibited by phage T7aiiA, resulting in a remarkable reduction in biomass by 74.9%, whereas the T7control phage only caused a reduction of 23.8% [63].

This design obviates the necessity of expressing, purifying, and delivering high doses of enzymes to hard-to-reach infection sites, thereby enhancing the effectiveness of phage therapy in biofilm removal. The cost-effectiveness of genome sequencing and synthetic biology technologies, such as phage genome refactoring and large-scale DNA synthesis, should facilitate the production of engineered enzymatic phages and expand the limited range of biofilm-degrading phages isolated from the environment.

### 3.5. Increasing the Half-Life of Phage In Vivo

Despite the clinical success of some antimicrobial treatments, the widespread application of phage therapies has been hindered by several severe physiochemical obstacles that phages encounter in the digestive or circulatory systems [64]. It is noteworthy that the efficacy of phages in vivo is decreased due to the action of phagocytes, antibodies, and gastric protease. In order to prevent the threat of phage abolition by the gastric acidic protease, an engineered phage T7 was constructed by fusing a membrane phosphoprotein (PhoE) signal peptide to the major capsid protein [65]. It interacted with the phospholipids of *E. coli* through the PhoE signal peptide, thus resulting in the formation of a lipid coating on the surface of T7 phage. The lipid-coated engineered T7 phage exhibited a 100-fold increase in stability compared to the wild-type phage T7 in the gastrointestinal tract of animals, representing a promising candidate for orally delivered phage therapy. Compared to other methods, such as microencapsulation, this approach offers the advantages of process simplicity, requiring significantly fewer optimization steps and a straightforward scale-up process (only the phage amplification is necessary). Importantly, this research demonstrates the feasibility, simplicity, and cost-effectiveness of phage engineering as a means of enhancing phage properties for oral administration in animals.

### 3.6. Reducing Endotoxin Release

Treatment with lytic phages can elicit rapid cell lysis, which may subsequently lead to the release of cell debris and toxins, thereby triggering an adverse immune response. To address this limitation, phages have been modified as nonreplicative variants. For instance, an engineered nonlytic phage (Pf3R) was constructed to minimize endotoxin release by replacing ORF40 with an endonuclease gene in the phage genome. The bactericidal efficacy of Pf3R remained unchanged, and it could effectively reduce the colony-forming units (CFUs) of *Pseudomonas aeruginosa* strain PAO1 by 99% upon infection, comparable to that of the parental phage [66]. However, the OD_600_ value of PAO1 infected with Pf3R remained constant for 7 h, and endotoxin levels in the supernatant were not significantly increased. In an animal trial, the survival rate of mice treated with a Pf3R phage was significantly higher than that of mice treated with a lytic phage. The improved survival rate observed in that study was associated with the attenuation of the inflammatory response induced by the Pf3R treatment, rather than the lytic phage itself.

The phage P954, which targets *Staphylococcus aureus*, was genetically modified to create a lysis-deficient variant (known as P954R) by replacing an endolysin-encoding gene with the chloramphenicol acetyl transferase (cat) gene through HR [1]. The phage P954R retained a genotype lacking endolysin and was capable of generating plaques by utilizing a heterologous endolysin that was expressed in the propagation host. The bactericidal efficacy of P954 and P954R was similar, both of which resulted in a nearly 90% reduction in colony-forming units (CFU). In addition, the engineered phage exhibited the ability to effectively treat lethal methicillin-resistant *Staphylococcus aureus* (MRSA) infections in mice, highlighting its potential as a promising therapeutic intervention.

These studies highlight the potential of endolysin gene disruption in reducing the number of phages released by their lytic parent phage following infection. In clinical settings, this approach offers the benefit of a precise dosage, addressing a key concern regarding phage therapy. Additionally, it may result in a reduced immune response and endotoxin release when targeting Gram-negative bacteria.

### 3.7. Engineering Phage as a Nanocarrier 

Phages can also be utilized as nanocarriers for the targeted elimination of pathogenic microorganisms and tumor cells through genetic manipulation or chemical modification [67]. These modified phages exhibit a specific recognition of targeted cells, triggering the controlled release of payloads. Peng et al. [68] engineered the filamentous phage M13 by exchanging its receptor-binding domain with that of another phage naturally targeting a different bacterial genus, resulting in the creation of a chimeric phage, M13KE. Subsequently, the pVIII shell proteins of the M13KE phage was chemically modified using n-succinimidyl S-acetylthioacetate (SATP) to introduce thiol groups, enabling the phage to conjugate with gold nanorods. Upon excitation with near-infrared light, the chimeric phage M13KE conjugated with gold nanorods released energy through nonradiative decay pathways, resulting in a localized heat generation that effectively eradicated the targeted bacterial cells. In addition, the irradiation of the gold nanorods resulted in the destruction of phages, reducing the potential toxicity compared to traditional phage therapy, while achieving a precise dosage control [69,70].

This approach proposed herein is most appropriate for treating tissues or surfaces that are directly accessible. In the short term, this technique could be used for localized topical therapy, particularly for wound infections or the colonization of medical devices, where engineered phages can be directly applied to the biofilm. For instance, *P. aeruginosa* is recognized as a pulmonary pathogen, but drug-resistant *P. aeruginosa* is also a significant pathogen in chronic wounds [46], surgical site infections, and burns [71]. These wounds are readily accessible for the application of therapeutic nanomaterials and NIR irradiation.

### 3.8. Clinical Application of Engineering Bacteriophages

In 2019, the application of genetically modified phages in clinical treatment was initially observed. In this particular instance, a 15-year-old recipient of a bilateral lung transplant was afflicted with an infection caused by *M*. *abscessus*, resulting in the development of cystic fibrosis in the transplanted lungs [25]. Three mycobacterophages, namely, Muddy, ZoeJ, and Bps, were administered intravenously to the patient as a therapeutic intervention. Two mycobacteriophages, ZoeJ and Bps, were genetically modified using the BRED strategy to eliminate the suppressor gene, resulting in their transformation into lytic bacteriophages. The administration of phages intravenously was well tolerated and resulted in a significant clinical improvement, such as the closure of sternal wounds, enhanced liver function, and a substantial resolution of infected skin nodules.

In a similar study, two types of mycobacteriophages, namely, D29_HRM^GD40^ and BPsΔ33HTH_HRM10, were administered intravenously to a 26-year-old patient suffering from severe cystic fibrosis caused by drug-resistant *M*. *abscessus* in pulmonary infections [72]. BPsΔ33HTH_HRM10 is a variant of the host range mutans (HRMs) derived from an engineered lytic derivative of BPs, and D29_ HRM^GD40^ is an HRM of D29 isolated on an *M. abscessus* strain GD40, which does not resemble the D29 parent’s effects on severe *M. abscessus* clinical isolates. The *M. abscessus* isolate was successfully eradicated through incubation with BPsΔ33HTH_HRM10 and D29_HRM^GD40^, alone or in combination, across a diverse range of bacterial and phage concentrations. Following the administration of the treatment, the patient’s lungs were effectively cleared of the drug-resistant mycobacterium abscesses, leading to the successful completion of a lung transplantation procedure. This case represents a significant milestone in the field of medical research as it marks the initial application of engineered phage therapy in the treatment of drug-resistant *M*. *abscessus*. 

However, the phages Muddy, BPs, and ZoeJ exhibited limited efficacy in eradicating other clinical isolates of *M. abscessus*, suggesting that the three-phage cocktail may not be universally effective. The intricate nature of cases such as this one poses challenges in accurately evaluating the efficacy of phage therapy. As there have been no prior reports of successfully treating pulmonary *M. abscessus* infection with phages, it is challenging to ascertain whether the outcomes observed in this singular case can be extrapolated to other patients undergoing this therapy. Additionally, there may be unidentified factors that have differentially influenced the treatment response.

**Table 1:** Bactericidal effect of genetically engineered phage.

Strain	Phage	Technology	Result	Ref.
*E. coli*	T3, T7	Engineering phage genomes in *Saccharomyces cerevisiae*	Expanding phage host range	[28]
*P. aeruginosa*	P793	Recombining with pGhost8	Expanding phage host range	[73]
*E. coli*	T2	Recombining with tail fiber gene	Expanding phage host range	[56]
*P. aeruginosa*	PaP1	Recombining with ORF84	Expanding phage host range	[74]
*E. coli*	T3	Phage tail fiber mutagenesis	Expanding phage host range	[75]
*E. coli*	Fd	Recombining with tail fiber gene	Expanding phage host range	[58]
*E. coli*	T2, Fd	Recombining with tail fiber gene	Expanding phage host range	[76]
*E. coli*	PSA	Recombining with receptor binding proteins (RBPs)	Expanding phage host range	[77]
*E. coli*	T4	Generating gp37 and gp38 variants	Expanding phage host range	[78]
*E. coli*	fd	Recombining with *OrfU*	Expanding phage host range	[59]
*E. coli*	T7	Recombining with *aiiA*	Reducing biofilm formation	[63]
*E. coli*	T7	Recombining with DspB	Reducing biofilm formation	[61]
*E. coli*	T7	Recombining with peptide 1018	Reducing biofilm formation	[79]
*P. aeruginosa*	Pf3	Recombining with endonuclease BglII	Reducing endotoxin production	[66]
*S. aureus*	P954	Recombining with chloramphenicol acetyl transferase (cat) gene	Reducing endotoxin production	[80]
*E. coli*	M13	Recombining with antimicrobial peptides (AMPs) and protein toxins	Reducing endotoxin production	[81]
*E. coli*	λ	Integrating with *Ndm-1* and *Ctx-M-15* using CRISPR/Cas	Restoring antibiotic sensitivity	[51]
*E. coli*	M13	Recombining with streptomycin sensitive genes	Restoring antibiotic sensitivity	[52]
*L. monocytogenes*	B025	Removing lysogen module	Improving lytic ability	[34]
*S. aureus*	φMN1	Integrating with CRISPR/Cas	Improving lytic ability	[48]
*S. aureus*	ØSaBov	Integrating with CRISPR/Cas	Improving lytic ability	[82]
*E. amylovora*	Y2	Recombining with Depolymerase	Improving lytic ability	[83]
*E. coli*	M13	CRISPR-cas9 target resistance genes and virulent genes	Improving lytic ability	[47]
*E. coli*	M13	Recombining with peptide RGD and PmpD	Improving lytic ability	[84]
*E. coli*	T4	HIV antigen was fused to outer capsid proteins	HIV vaccine	[85]
*E. coli*	T4	Anthrax toxin proteins was fused to outer capsid proteins	Anthrax vaccine	[86]
*E. coli*	T4	FMDV p1 protein was fused to outer capsid proteins	FMDV vaccine	[87]
*E. coli*	MS2	Capsids radiolabeled with ^64^Cu	Targeted drug carriers	[88]
*E. coli*	λ	Recombining with integrin-binding peptide	Phage-mediated gene delivery and expression	[89]
*S. typhimurium*	P22	Chemical modification by DTPA	Gd (III) carrier	[90]
*E. coli*	T7	Recombining with gold-binding peptide	Gold nanorods carrier	[91]
*E. coli*	fd–tet	Self-assembled siRNA−nanophages	siRNA carrier	[92]
*E. coli*	M13	Chemical modification to form Au-S bonds	Gold nanorods carrier	[68]
*E. coli*	M13	Recombining with a biotin acceptor peptide (BAP)	Targeted drug carriers	[93]
*E. coli*	M13	Recombining with a biotin acceptor peptide (BAP)	Targeted drug carriers	[94]
*E. coli*	fUSE5-ZZ	Recombining with IgG Fc-binding ZZ domain of protein A	Targeted drug carriers	[95]
*E. coli*	fUSE5-ZZ	Recombining with IgG Fc-binding ZZ domain of protein A	Antibacterial drug carriers	[96,97]
*E. coli*	f88	Recombining with myelin oligodendrocyte glycoprotein (MOG)	Vector-mediated antigen delivery	[98]
*E. coli*	T4	Recombining with GFP	Luciferase reporter phage	[99]
*E. coli*	T7	Recombining with biotinylation peptide	Streptavidin-coated quantum dots reporter phage	[100]
*B*. *anthracis*	Wβ	Recombining with *luxAB-2*	Bioluminescent reporter phage	[101,102]
*E. coli*	phiV10	Recombining with luxCDABE operon	Luciferase reporter phage	[103]
*L. monocytogenes*	A511	Recombining with nanoluciferase	Nanoluciferase (NLuc) reporter phage	[104]
*E. coli*	T7	Recombining with nanoluciferase	Nanoluciferase (NLuc) reporter phage	[105]
*E. coli*	ΦV10	Recombining with nanoluciferase	Nanoluciferase (NLuc) reporter phage	[106]
*E. coli*	K1E	Recombining with nanoluciferase	Nanoluciferase (NLuc) reporter phage	[107]
*E. coli*	T7	Recombining with β-galactosidase	β-galactosidase reporter phage	[108]
*M. smegmatis*	TM4	Recombining with GFP or ZsYellow	Fluorescent reporter phage	[109]
*M. smegmatis*	D29	Recombining with Phsp60-egfp cassette using BRED	EGFP reporter phage	[24]
*E. amylovora*	Y2	Homologous recombination with LuxAB	Luciferase reporter phage	[83]
*E. coli*	T7	Homologous recombination with PhoE	Enhancing the half-life of phage	[65]
*L. monocytogenes*	A511	Bacteriophages PEGylation	Enhancing the half-life of phage	[110]
*S. typhi*	Felix-O1	Bacteriophages PEGylation	Enhancing the half-life of phage	[110]
*E. faecalis*	fEf11	Recombining with defective prophage	Improving lytic ability and expanding phage host range	[111]

## 4. Discussion

Compared to natural phages, genetically modified phages offer stronger advantages in combating bacterial infections. As stated in Section 3, genetically modified phages have demonstrated an enhanced efficacy in combating bacterial infections through various mechanisms, including an improved bactericidal activity, restored antibiotic susceptibility, decreased endotoxin secretion, and other related effects. Additionally, genetically modified phages have the ability to display antigenic peptides on their capsid proteins, thereby effectively activating the immune system and eliciting immune responses. For instance, phage T4 has been demonstrated to effectively present HIV and anthrax toxin antigens on its surface, thereby eliciting an optimal immune response in mice [86,112]. Finally, genetically modified phages are eligible for patent protection, whereas natural phages are not. This enables companies that have utilized phages as products to attract investment in the capital market, thereby promoting the advancement of engineered phage research [4].

While engineered phages offer numerous benefits, there are also potential consequences that need to be considered. Firstly, there is no doubt that the safety of utilizing engineered phages in human applications is of paramount concern. While phages are generally regarded as safe, rigorous testing is imperative to ensure that engineered phages do not elicit any deleterious effects on the overall health of patients. Additionally, like antibiotics, phages can also develop tolerance in host bacteria. Furthermore, there are concerns regarding the potential for phages to trigger bacterial tolerance similar to that observed with antibiotics. While certain treatment approaches, such as cocktail therapy, have been shown to mitigate bacterial tolerance to phages, the sustained efficacy of phage therapy may still be constrained by the existence of phage tolerance [113]. Finally, in comparison to conventional antibiotics, the regulatory framework for phage therapy remains imperfect. The approval process for phage therapy may be more intricate and time-consuming, potentially impeding patients in need from accessing this treatment modality.

While there are numerous unresolved issues surrounding the application of engineered phages, it is indisputable that, given the pervasive issue of antibiotic resistance, they continue to represent a crucial area of current research. Engineered phages possess immense potential for diverse applications, owing to their ability to be designed and modified to meet specific requirements for each application. Hence, they are emerging as a novel category of biological agents with extensive potential.

## Data Availability

The study did not report any data.
